# Synergistic Anti-Cancer Activity of Melittin and Erlotinib in Non-Small Cell Lung Cancer

**DOI:** 10.3390/ijms26072903

**Published:** 2025-03-22

**Authors:** Hairulislam M. Ibrahim, Jihad Alessa, Hala Badr Khalil, Gamal A. Bekhet, Ashraf Khalifa

**Affiliations:** Biological Science Department, College of Science, King Faisal University, P.O. Box 400, Al-Ahsa 31982, Saudi Arabia

**Keywords:** lung cancer, Melittin, Erlotinib, JAK2/JAK3 signaling, synergistic anti-cancer effects

## Abstract

Lung cancer remains a leading cause of cancer-related mortality worldwide. Despite advancements in current therapies, the development of drug resistance and the need for improved treatment outcomes necessitate the exploration of novel therapeutic approaches. This study aimed to investigate the synergistic anti-cancer effects of Melittin, a bee venom peptide, in combination with Erlotinib, an EGFR inhibitor, in non-small cell lung cancer (NSCLC). The study evaluated the combined effects of Melittin and Erlotinib on A549 NSCLC cells. Cell viability, proliferation, migration, and apoptosis were assessed using standard in vitro assays. Mechanistic studies investigated the impact of the combination treatment on key signaling pathways, including those involving JAK2 and JAK3. Molecular docking simulations were performed to predict the binding interactions between Melittin and these kinases. The combination of Melittin and Erlotinib significantly inhibited A549 cell proliferation and migration, with a marked reduction in cell viability and enhanced apoptosis compared to either agent alone. Mechanistically, Melittin demonstrated interactions with JAK2 and JAK3, key proteins involved in apoptotic signaling. Molecular docking simulations further supported these findings, predicting strong binding affinities between Melittin and both kinases. These findings demonstrate a synergistic anti-cancer effect of Melittin and Erlotinib in A549 NSCLC cells. The observed interactions with JAK2 and JAK3 suggest a potential mechanism for Melittin’s activity. These results highlight the potential of Melittin as a promising adjuvant to Erlotinib for the treatment of NSCLC.

## 1. Introduction

Lung cancer remains one of the most challenging malignancies to treat, necessitating the exploration of novel therapeutic approaches. Despite significant advancements in conventional treatments, such as surgery, chemotherapy, and radiation therapy, lung cancer continues to exhibit high mortality rates [1]. Lung cancer treatment faces challenges like drug resistance, severe side effects, and limited options for advanced stages. This underscores the urgent need for new therapies that effectively target cancer cells while minimizing harm to healthy tissues. Non-small cell lung cancer (NSCLC) is the most prevalent type of lung cancer, comprising approximately 80–85% of all cases. It is distinguished from other lung cancers, particularly small cell lung cancer, by the appearance of its cells under a microscope [1].

In this context, Melittin, a major component of bee venom, has emerged as a promising anti-cancer agent with demonstrated efficacy in preclinical models [2]. Its potent cytotoxic effects, primarily through the disruption of cellular membranes and induction of apoptosis, offer a potential alternative or adjunct to existing treatments.

Understanding Melittin’s anti-cancer mechanisms is crucial. This understanding is needed to translate its preclinical success into effective and safe therapies, especially for the challenges facing traditional NSCLC treatments. [3].

A key aspect of Melittin’s anti-cancer action involves its interactions with critical cellular proteins, particularly those involved in regulating programmed cell death pathways. Previous studies have shed light on the structural and functional aspects of these interactions. For instance, research has elucidated the structural features of Melittin and its ability to interact with key proteins involved in apoptosis, including members of the Bcl-2 family, caspases, and death receptors (DRs) [4]. Furthermore, a seminal study provided insights into Melittin-protein complexes. It revealed Melittin’s flexibility and its ability to change shape when binding to different proteins [5]. These dynamic interactions highlight the multifaceted nature of Melittin’s activity and its ability to modulate various apoptotic pathways.

Computational approaches have significantly advanced our understanding of these molecular interactions. Molecular docking simulations have proven invaluable in predicting binding modes and identifying key residues involved in Melittin-protein interactions, thus providing crucial insights into the molecular basis of its pro-apoptotic effects [6,7]. These studies have demonstrated Melittin’s ability to modulate various apoptotic pathways in cancer cells, including the intrinsic mitochondrial pathway and the extrinsic death receptor pathway [8]. Importantly, computational analyses have revealed Melittin’s potential to disrupt critical protein–protein interactions within these pathways, leading to the activation of apoptosis and inhibition of cancer cell proliferation [9,10].

This study aims to further investigate the molecular mechanisms of Melittin’s anti-cancer activity by focusing on its interactions with key proteins involved in cancer-related signaling pathways. Specifically, we investigate the potential interactions of Melittin with five critical programmed cell death proteins: Janus kinase 2 and 3 (JAK2 and JAK3), and signal transducer and activator of transcription 3 (STAT3). These proteins play pivotal roles in various cancers, including lung cancer, by regulating cell growth, survival, and inflammation.

## 2. Results

### 2.1. Evaluation of the Effect of Melittin and Erlotinib on A549 Lung Cancer Cells

**Figure 1:**
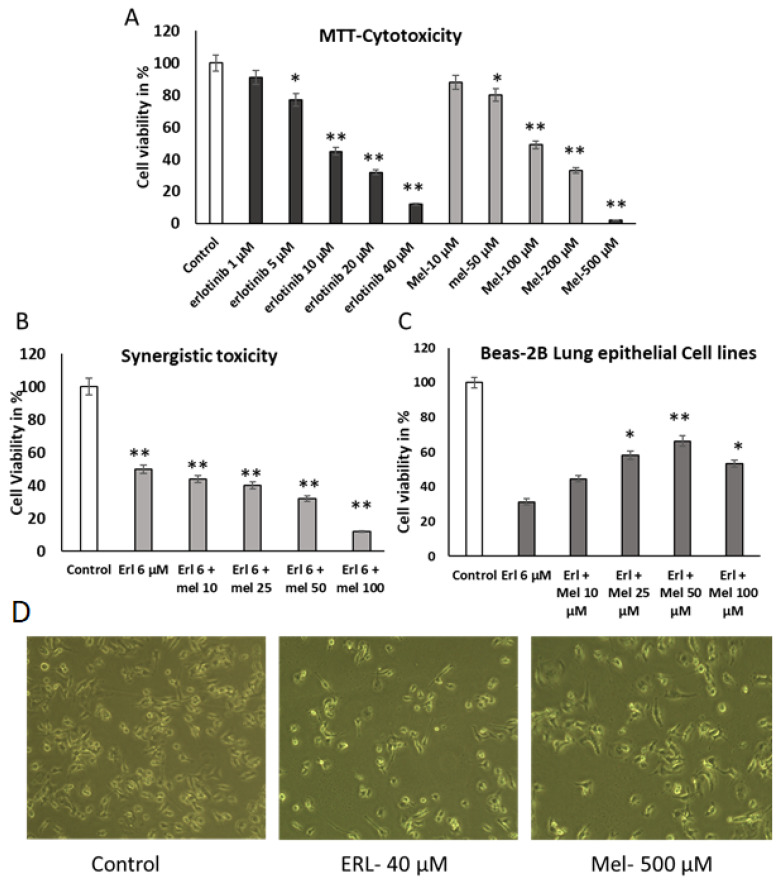
Effect of Melittin and Erlotinib on the growth of A549 lung cancer cells and Beas-2B lung epithelial cell line. (**A**) Cell viability was determined by SRB assay. (**B**,**C**) Effect of Melittin and Erlotinib on the cell viability in A549 cells and Beas-2B. (**D**) Phase contrast images of treated A549 cells at 20× magnification. Data were expressed as the mean ± S.D. of three experiments. * *p* < 0.05 and ** *p* < 0.01 indicate statistically significant differences from control group.

This study investigated the anti-proliferative effects of Melittin and Erlotinib on A549 human lung cancer cells. Cell viability assays (SRB) demonstrated significant dose-dependent growth inhibition by both agents, with IC50 values of 50 µM and 6 µM for Melittin and Erlotinib, respectively (Figure 1A). This growth inhibition was further supported by a significant reduction in colony formation in A549 cells treated with both agents (Figure 1B), indicating suppression of their proliferative capacity.

Morphological examination using phase-contrast microscopy revealed pronounced changes in A549 cells treated with Melittin and Erlotinib. Treated cells exhibited characteristic features of apoptosis, including cell shrinkage, membrane blebbing, and detachment from the substratum, compared to untreated controls (Figure 1C). These findings suggest that the observed growth inhibition results from the induction of apoptosis in A549 lung cancer cells. These morphological alterations are characteristic of apoptotic cell death and are consistent with the observed decrease in cell viability and colony formation.

### 2.2. Melittin Abrogates A549 Migration Using Scratch Wound Assay

**Figure 2:**
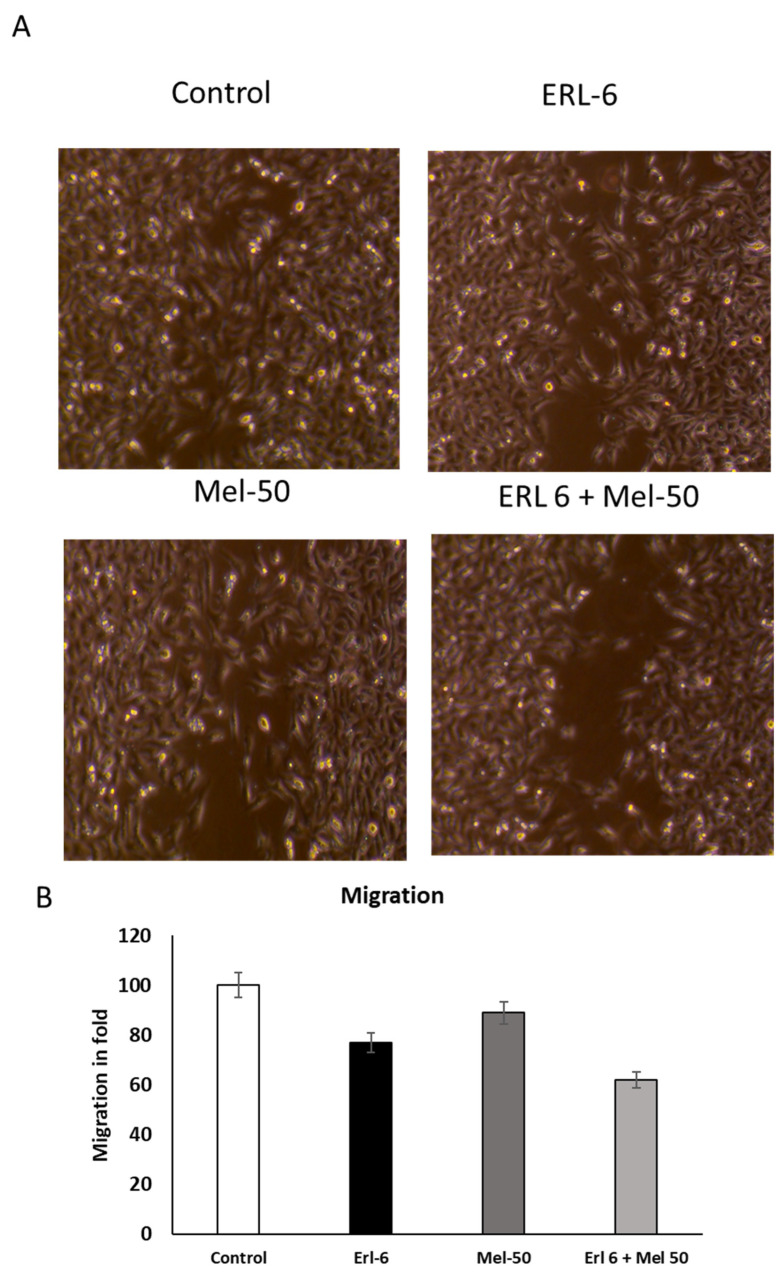
Effect of Melittin and Erlotinib on the cell migration (wound healing activity) of A549 lung cancer cells. After 50% cell confluent in a 12-well plate, a scratch was made by using sterile tips in the middle of the well and treating the plate with Melittin 50 µM and Erlotinib 6 µM concentrations, respectively (**A**) Effect of Melittin and Erlotinib on the cell migration and wound healing activity for A549 cells and phase contrast images of treated cells at 20× magnification. (**B**) Cell migration was recorded in fold changes. Data were expressed as the mean ± S.D. of three experiments.

To investigate the impact on cell migration, A549 cells were treated with partial toxic concentrations of Erlotinib (6 µM) and Melittin (50 µM). Both Erlotinib and Melittin individually inhibited A549 cell migration in a concentration-dependent manner (Figure 2A). Notably, the combination of Erlotinib and Melittin demonstrated a significantly stronger suppression of A549 cell migration compared to either drug alone, whereas Melittin at 50 µM had no discernible effect on cell migration (*p* < 0.05) (Figure 2B).

These findings, consistent with results from the scratch wound assay, demonstrate that both Erlotinib and Melittin exhibit inhibitory effects on the migration of A549 lung cancer cells. While Erlotinib alone significantly suppressed cell migration, Melittin at this concentration showed no discernible effect. However, the combination of Erlotinib and Melittin exhibited a synergistic inhibitory effect on cell migration, significantly surpassing the effect of either drug alone. These findings suggest that the combination of Erlotinib and Melittin may have a potent anti-metastatic effect by effectively inhibiting the migratory capacity of lung cancer cells

### 2.3. Melittin Upregulates Mitochondrial Mediated Apoptotic Markers

**Figure 3:**
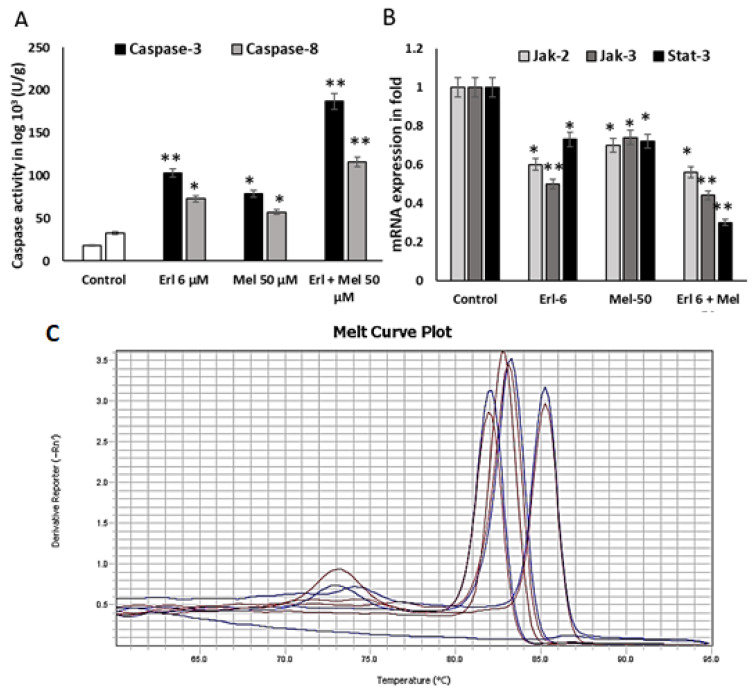
Effect of Melittin and synergistic effect on the expression of apoptosis regulatory markers. (**A**). Apoptotic activity of Melittin and Erlotinib on downstream apoptotic markers Caspase-3 and caspase-9 using the Elabscience colorimetric kit assay. (**B**). Expression of apoptosis regulatory proteins related intrinsic pathway was determined by quantitative PCR of JAK-2, Jak-3 and Stat-3 with GAPDH internal control. (**C**). Melting curve of qPCR expression values under expression fold indicate the cT values. Blue curve indicate Jak-2, Yellow curve indicate Jak-3, Red curve indicate State-3 and Violet curve indicate GAPDH. Data were expressed as the mean ± S.D. of three experiments. * *p* < 0.05 indicates statistically significant differences between groups and ** *p* < 0.01 represents significance between the groups.

Caspase-3 and 8 activity were estimated using caspase specific substrate catalytic activity and determined the Melittin induced apoptosis. These experiments were performed three times independently. Melittin (50 μM) was incubated for 3 h with A549 cells, then cell lysate was used to detect the caspase-3 activity, and the absorbance at 405 nm was measured using a microplate reader. The caspase-3 activity was significantly higher after exposure to treatment for 3 h compared to the control (18.3 ± 1.1, 79.20 ± 3.3, and Erlotinib expressed 103.1 ± 4.1 U/g of protein), whereas the synergistic effect showed 187.3 ± 3.56. With the addition of the caspase-8 activity, there was comparatively less catalytic activity than with caspase-3. These showed 57.1 ± 1.7, 73.1 ± 4.1 and 117 7 ± 3.1, respectively (Figure 3A). The values for caspase-8 showed a significant different between the Melittin and control groups and the synergistic group.

### 2.4. Melittin Downregulates Cancer Progressive Genes

Melittin downregulates cancer-progressive genes by inhibiting the JAK2/STAT3 pathway. Melittin treatment significantly inhibited the phosphorylation of JAK2 and STAT3 (Figure 3A). Since Erlotinib, a known inhibitor of JAK2, also dramatically decreased STAT3 phosphorylation, it is plausible that the inhibition of JAK2 activity contributes to the reduction of STAT3 activation by Melittin (Figure 3B). The suppression of STAT3 mediated by Melittin was further supported by the observed inhibition of JAK2 and JAK3 activity (Figure 3). Inhibiting the JAK2/JAK3 pathway and subsequently inactivating STAT3 demonstrated a synergistic effect when combined with Erlotinib treatment. This combined treatment successfully suppressed the expression of key cancer-progressive markers, thereby inhibiting cell proliferation and growth. Overall, these findings suggest that Melittin significantly enhances the activity of Erlotinib against lung cancer cell lines by modulating the JAK2/STAT3 signaling pathway.

### 2.5. To Elucidate Potential Molecular Targets of Melittin, We Conducted In Silico Protein–Protein Docking Simulations Focusing on Key Regulators of Apoptotic Signaling Pathways, Specifically JAK2, JAK3, and STAT3

**Figure 4:**
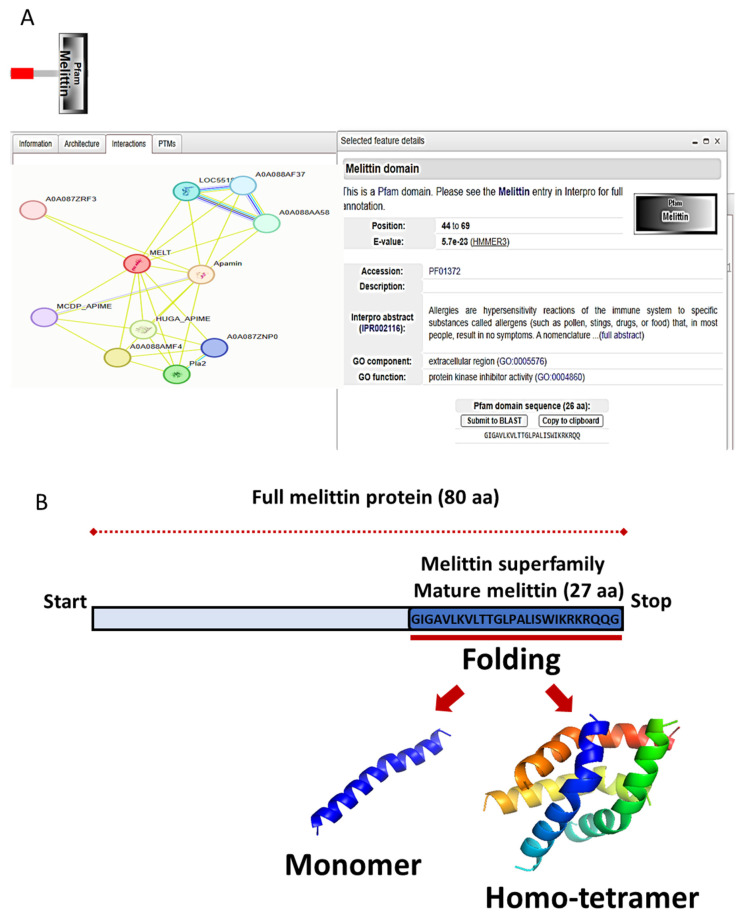
Melittin interaction and virtual eray structure analysis. (**A**) Protein network analysis and its interaction with other protein was revealed using protein-protein interaction tool. (**B**) The 3D fold of mature Melittin protein. Illustration of the protein models folded using AlphaFold v2, including the monomeric conformation of Melittin. Additionally, a homo-tetrameric structure of Melittin is revealed through X-ray analysis.

We utilized the LZerD docking algorithm to investigate the binding interactions and affinities between Melittin and these proteins. Prior to docking, high-quality structural models for each protein were generated. The mature Melittin peptide sequence (GenBank accession: AFI40556.1) was predicted to adopt a characteristic helix–hinge–helix motif using AlphaFold (Figure 4).

**Figure 5:**
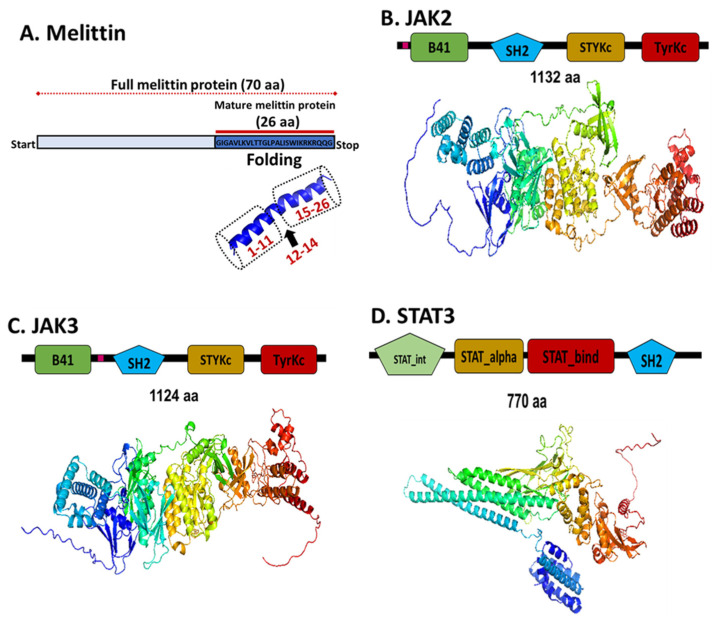
Domain architecture and structural folding of Melittin, JAK2, JAK3, and STAT3. (**A**). Melittin: The full protein sequence 70 aa; the mature 26–amino acid peptide adopting a helix–hinge–helix motif; N–terminal region involves a helix–hinge–helix motif, where residues 1–11 form an α–helix, followed by a hinge region (residues 12–14), and another α–helix spanning residues 15–26, C–terminal region. (**B**). JAK2: Domain structure featuring B41 domain, SH2 (Src Homology 2) domain, pseudo–kinase domain (STyKc), and tyrosine kinase domain (TyrKc). (**C**). JAK3: Similar domain organization to JAK2, with approximately 50% sequence identity. Domains include FERM, SH2, STyKc, and TyrKc. (**D**) STAT3 exhibits the N–terminal domain (STAT_int), the coiled–coil domain (STAT_alpha), the DNA–binding domain (STAT_bind), and the Src Homology 2 (SH2) domain. Protein structures were predicted using SWISS–MODEL and AlphaFold. Domain boundaries are indicated by different colors.

For the target proteins (JAK2: GB: NP_001309125.1, JAK3: GB: AAC50950.1, STAT3: GB: NP_644805.1), reliable secondary structure models were constructed using the SWISS–MODEL pipeline, incorporating template identification, target–template alignment, model building, and rigorous quality estimation (Table 1; Figure 4 and Figure 5).

Pairwise protein–protein docking simulations were performed between Melittin and each of the three human protein structures using the LZerD algorithm. Binding affinities between Melittin and the investigated proteins were assessed using GOAP, DFIRE, and IT scoring functions (Figure 4 and Figure 5). Proteins were ranked based on a composite score derived from these metrics (Table 2). Top-ranked docked complexes, determined by LZerD ranksum, were then clustered using RMSD. The analysis revealed intriguing insights into the interaction profiles of specific proteins with Melittin. Notably, JAK3 demonstrated particularly robust affinity, as evidenced by their favorable ranksum of about 19. This suggests a strong potential for specific binding interactions between Melittin and JAK3 with an acceptable RMSD. In the same line, JAK2 exhibited less pronounced affinity rank, and displayed the most favorable RMSD (3.2) to Melittin.

**Figure 6:**
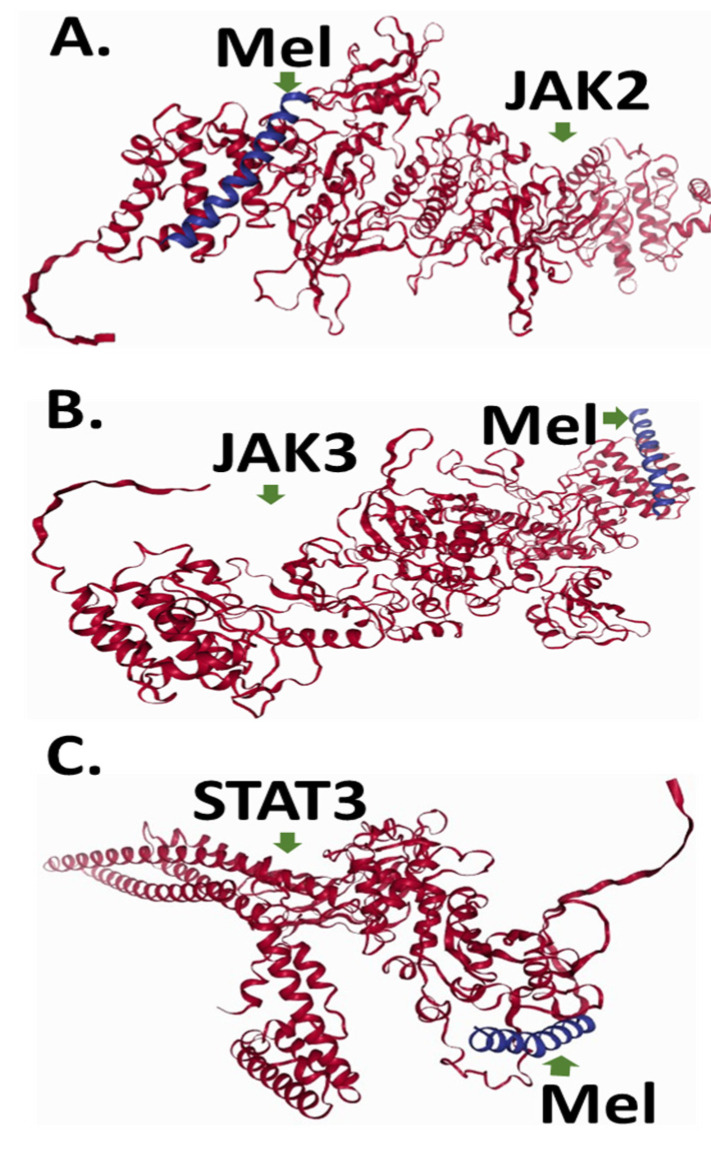
Molecular docking of Melittin–human protein interactions. Representation of the 2D interaction graph depicting (**A**). Mel–JAK2 complex, (**B**). Mel–JAK3 complex; (**C**). Mel–STAT3 complex. In the graph, red color denotes human proteins, while blue represents Melittin. In the graph, red colour denotes human programmed cell death proteins, while blue represents Melittin in a monomer stat.

Sequence alignment analysis revealed that JAK2 and JAK3 share approximately 50% amino acid identity, indicating a significant degree of structural and functional similarity. Both proteins exhibit a conserved domain architecture characteristic of the JAK family, which includes the FERM (B41) domain, Src Homology 2 (SH2) domain, pseudo-kinase domain (STyKc), and tyrosine kinase domain (TyrKc) (Figure 4 and Figure 5). These domains are critical for mediating protein–protein interactions, signal transduction, and kinase activity, which are essential for their roles in the JAK–STAT signaling pathway. In contrast, STAT3 exhibited the lowest binding affinity to Melittin in the docking simulations. The observed variations in docking scores and RMSD values between Melittin and its interacting partners may reflect differences in their binding interfaces, conformational flexibility, and electrostatic complementarity (Figure 6). For instance, the robust affinity of JAK3 to Melittin, as evidenced by its favorable ranksum score (~19) and acceptable RMSD, suggests a strong potential for specific molecular interactions. Similarly, while JAK2 displayed a less pronounced affinity rank, it exhibited the most favorable RMSD (3.2 Å) with Melittin, indicating a high degree of structural compatibility. These findings highlight the versatility of Melittin in interacting with both JAK2 and JAK3, despite their differences in binding affinities and structural features. The results suggest that Melittin may engage with these proteins through distinct molecular mechanisms, potentially influenced by variations in their domain organization, surface properties, and dynamic behavior. Further analysis of the binding interfaces and interaction hotspots could provide deeper insights into the specific residues and structural motifs driving these interactions.

The observed variations in docking scores and RMSD values likely reflect differences in the binding interfaces, conformational flexibility, and electrostatic complementarity between Melittin and each target protein. These findings suggest that Melittin may interact with both JAK2 and JAK3 through distinct mechanisms, potentially impacting their downstream signaling and contributing to Melittin’s anti-cancer effects.

## 3. Discussion

Melittin, a potent antimicrobial peptide derived from bee venom, has emerged as a promising anti-cancer agent with demonstrated efficacy against various cancer cell lines, including A549 lung cancer cells [11]. Its anti-cancer properties primarily stem from its ability to disrupt cellular membranes, leading to increased permeability and ultimately cell death. This cationic, amphipathic peptide interacts with the lipid bilayer, forming pores that destabilize the membrane and induce cell lysis [11,12]. Beyond its membrane-disrupting effects, Melittin has been shown to modulate crucial signaling pathways involved in cancer progression, including proliferation, apoptosis, metastasis, and angiogenesis [12].

Melittin may have anti-cancer effects. It may achieve this by changing the signals, genes, and molecules involved in cancer processes. Specifically, Melittin has been shown to target the Janus kinase-signaling pathway, which plays a crucial role in the regulation of cell growth, differentiation, and survival. The JAK–STAT signaling axis has been implicated in the proliferation and survival of various cancer cells and may even contribute to resistance mechanisms against certain targeted therapies [13]. Jak2 signaling, in particular, has been linked to the progression of certain cancer types, making it a promising target for therapeutic intervention [13].

Mitochondria-derived activator of caspase leads to apoptosis. Smac affinity may promote caspase activation by binding to apoptosis proteins to inhibit its activity, giving rise to relief of caspase-binding partners and abduction of cell apoptosis [14]. In this study, caspase cascade protein levels were accompanied by cell apoptosis as the Melittin concentration was increased (Figure 3A). It is known that Melittin can induce caspase-3 activation, and caspase-3 can degrade anti-apoptotic markers.

The observed differential binding affinities of Melittin for JAK2, JAK3, and STAT3 suggest a preferential targeting of the JAK–STAT signaling pathway. This pathway plays a pivotal role in regulating cell growth, differentiation, and survival, and its aberrant activation is a hallmark of numerous cancers. Notably, JAK2 and JAK3 are important regulators of STAT3. STAT3 is a transcription factor that drives oncogenic processes, such as proliferation, survival, angiogenesis, and metastasis. The JAK–STAT pathway is a critical signaling pathway involved in various cellular processes, but its persistent activation is implicated in numerous diseases. Understanding the intricate mechanisms of JAKSTAT signaling in disease formation is crucial for developing effective therapeutic strategies [5].

The in silico analysis conducted in this study provides insights into Melittin’s potential protein targets. This study uniquely explores Melittin’s interactions with JAK2, JAK3, and STAT3, which are crucial components of breast cancer signaling pathways [6]. The results indicate that Melittin exhibits differential binding affinities to these apoptosis-related proteins, with JAK3 showing the strongest interaction, followed by JAK2, then STAT3. This hierarchical binding preference suggests that Melittin may preferentially target specific components of the JAK–STAT signaling pathway, potentially leading to pathway disruption and subsequent apoptosis induction. The robust affinity of Melittin for JAK3, as evidenced by its favorable ranksum score (~19) and acceptable RMSD, highlights its potential as a selective modulator of JAK3 activity. Conversely, JAK2, which displayed the most favorable RMSD (3.2 Å) with Melittin despite a lower affinity rank, may engage with Melittin through structurally complementary interfaces. The conserved domain architecture of JAK2 and JAK3, including their FERM, SH2, pseudo-kinase, and tyrosine kinase domains, likely provides shared structural motifs that facilitate Melittin binding. However, the binding scores and RMSD values show differences. These differences suggest subtle mechanistic distinctions, possibly due to variations in electrostatic complementarity or conformational flexibility. The 50% amino acid identity between JAK2 and JAK3, coupled with their conserved domain organization, provides a structural basis for their shared affinity with Melittin. However, differences in binding scores suggest that non-conserved residues or isoform-specific surface features (e.g., charge distribution or loop conformations) may influence the strength and specificity of these interactions. For instance, JAK3’s strong ranksum score could reflect unique electrostatic or hydrophobic patches in its kinase domain that favor Melittin binding. The findings indicate that the fusion protein is stable, exhibits strong interaction potential, and has a high expression feasibility, suggesting its promise as a therapeutic drug candidate [10,15].

Melittin shows a strong binding affinity to JAK2–JAK3. This suggests that Melittin may interact directly with these kinases, possibly inhibiting their activity. This inhibition could lead to a downstream suppression of STAT3 phosphorylation and activation, ultimately disrupting critical cancer-promoting signaling pathways [16,17]. The weaker interaction between Melittin and STAT3 aligns with its divergent structural and functional roles in the JAK–STAT pathway. STAT3 primarily acts as a transcription factor downstream of JAK activation, and its reduced affinity for Melittin may reflect differences in surface-exposed residues or binding interfaces compared to the kinase domains of JAK2–JAK3. This selectivity underscores Melittin’s potential to preferentially target upstream signaling components (JAKs) rather than downstream effectors (STATs). This reveals the minimization of off-target effects in therapeutic contexts. Future studies should focus on experimentally validating the predicted interactions between Melittin and JAK2/JAK3/STAT3. This could involve in vitro binding assays, co-immunoprecipitation experiments, and functional studies to assess the impact of these interactions on JAK–STAT signaling. It has been reported that encapsulating ligand loaded PLGA nanoparticles enhances drug stability, supports targeted delivery, and allows for sustained release. This study highlights the therapeutic potential of Melittin with Erlotinib in targeting the JAK2/STAT3/mTOR/PI3K pathways for A549 lung cancer treatment. Further research is necessary to clarify the underlying mechanisms and assess the clinical applications of this novel immunotherapy strategy.

Furthermore, the observed differential binding affinities may reflect distinct binding modes and potential allosteric effects of Melittin on these kinases [18,19]. This suggests that Melittin may not simply inhibit kinase activity but could also modulate its function in more complex ways, such as altering substrate specificity or interacting with regulatory proteins. Recent advancements, including sequence modifications, genetic engineering, and nano-delivery systems, aim to overcome these limitations by enhancing selectivity and reducing toxicity. These innovations promise to expand the therapeutic potential of Melittin, particularly in cancer treatment, by improving its efficacy and safety [20].

Molecular docking is a powerful tool for predicting protein–ligand interactions, but it has inherent limitations that require experimental validation. Docking relies on static protein structures and does not account for dynamic conformational changes, solvent effects, or allosteric modulations that may influence binding affinity. Additionally, docking scores indicate potential binding but do not confirm functional inhibition, as factors like post-translational modifications and intracellular localization can affect biological activity. To validate our findings, future studies should incorporate molecular dynamics simulations for interaction stability, surface plasmon resonance for binding affinity measurements, and kinase activity assays to assess JAK2/JAK3 inhibition. These experimental approaches will be essential to confirm Melittin’s predicted effects on JAK–STAT signaling and its potential as a therapeutic agent.

These findings have significant implications for the development of novel anti-cancer therapies. Melittin, with its potential to directly target key signaling proteins involved in tumorigenesis, may offer a promising therapeutic avenue, particularly in combination with existing therapies that target the JAK–STAT pathway. However, further experimental validation is crucial to confirm these in silico findings and elucidate the precise molecular mechanisms underlying Melittin interactions with JAK2, JAK3, and STAT3. It is worth mentioning that this study shows that Melittin can boost the anti-cancer effects of Erlotinib in NSCLC, potentially by affecting the JAK2–JAK3 pathway, which could be helpful even in KRAS-mutant cancers. However, the study used A549 cells, which have a KRAS mutation and do not rely on EGFR, the target of Erlotinib. While these cells are often used in research, they are not ideal for studying Erlotinib’s effects specifically. Therefore, while the study highlights the synergistic potential of the combination and its mechanism, the results concerning Erlotinib’s individual activity should be addressed cautiously when considering EGFR-driven cancers. Specifically, the observed synergy may be mediated through mechanisms independent of EGFR signaling in the A549 cell line. Future research using EGFR-dependent models will be necessary to fully confirm the clinical relevance of this combination, especially for EGFR-mutant NSCLC, and to determine the precise contribution of EGFR inhibition to the observed synergistic effect. Additionally, the importance of in vivo validation is acknowledged and emphasized as a critical direction for future research.

## 4. Materials and Methods

### 4.1. Cell Line Sources

A549 cell lines were obtained from KFSHRC, Saudi Arabia and HEK293FT cell lines purchased from Pondicherry Centre for Biological sciences, India. Cells were incubated at 37 °C in a humidified atmosphere containing 5% CO_2_ and supplemented with 1% antibiotic–antimitotic. HEK293FT cells (human embryonic kidney 293 cells stably were cultured in DMEM with 10% FBS, supplemented with 1% glutamine and 0.4 mg/mL G418 Genetic (Gibco, Darmstadt, Germany).

Preparation of Melittin and Erlotinib solution. Melittin was dissolved in 90% RPMI and 10% ethanol *v*/*v* in media, under aseptic conditions. Treatments of cells with Melittin were performed as per indicated concentration(s); however, cells were treated with Melittin and Erlotinib 30 min after seeding the cells in the case of the combination.

Treatment protocol. A549 and Hek293 cells were treated with different concentrations of Melittin and Erlotinib (ranging from 10 µM to 500 µM and 1 µM to 40 µM, respectively), to discover the cell inhibitory concentrations.

Cytotoxicity studies using Sulforhodamine B assay. Exponentially growing (3 × 10^3^) cells were seeded and incubated for proper attachment on surface (in a 96-well plate), and thereafter cells were treated with increasing concentrations of Melittin and Erlotinib ranging from 10 µM to 500 µM and 1 µM to 40 µM, respectively, in a simultaneous model using a serial dilution process. The treatment was made in triplicate wells. After treatments, cells were washed with phosphate buffered saline, and fixed using 10% TCA. Plates were air-dried and cells were stained with 0.4% SRB in 1% acetic acid (*w*/*v*) in milli Q. After staining of cells, plates were washed three times with PBS, and SRB extracted using extraction buffer (10 mM Tris base solution (*w*/*v*); pH 10.5). The absorbance of extracted dye was recorded by spectrophotometer (BIOTEK, Vermont, USA) at λ565 nm and λ690 nm as reference wavelength.

### 4.2. Wound Healing Assay (Scratch Wound Assay)

To study cell migration, A549 and Hek293 cells were seeded at 5–10^5^ cells/well in 24-well plates and incubated until they reached 70% confluence [21]. Monolayer cells were scratched with a 200 μL sterile tip to create a wound, and cells were then washed twice with serum-free culture media to remove floating cells. Media were replaced with fresh serum-free medium. Cells were incubated in IC50 concentration of Melittin and Erlotinib in 50 µM and 6 µM, respectively, in the medium for 24 h and each treatment was made in triplicate wells and experiments were repeated three times. Treated cells were washed with PBS, fixed, and microscopic photos were made after 24 h and recorded the migration value in fold.

### 4.3. Caspase-3 and Caspase-8 Activity Detection

The activity of caspase-3 and caspase-8 were determined using caspase-3 and caspase-8 activity assay kits (Elabscience, Wuhan, China) [22]. Briefly, A549 lung cancer cell lines were cultured in 12 well plate (1 × 10^4^ cells/well) and allowed to attach overnight. After treatment with Melittin, 50 μM was incubated. Cell lysate was prepared with 100 μL lysis buffer for 15 min on ice. The cell lysates were centrifuged at 13,000× *g* for 15 min at 4 °C. Cell-free supernatant was collected and stored for further use. Add 10 μL Ac-DEVD-pNA and the sample solution for each group, containing 75 μL reaction buffer; a 15 μL sample was incubated in a 96-well microplate for 3 h at 37 °C. The caspase-3 and caspase 8 activity was measured at 405 nm using a microplate reader. According to the protein standard, we generated a standard curve (Y = −22.7602 + 420.9950X, R = 0.9900), and the caspase-3 activity units (U/g) of the experimental groups and the control groups were calculated. These experiments were performed three times, independently.

### 4.4. Gene Expression Analysis by Real-Time PCR

Up to 1 × 10^6^ cells were cultured in T25-flasks, and treated with IC50 concentrations of Melittin, and ERL concentration at 50 µM and 6 µM, respectively, for 48 h. Add 1 mL ice-cold TRIZOl solution to a 2 mL tube containing cell homogenate. Total RNA was extracted and quantified using nanospec. Mix the template RNA (total RNA or Poly (A) mRNA) 1 ng–5 μg, Buffer-Mix (2X) 10 μL, Enzyme-Mix 2 μL, and DEPC-treated water up to 20 μL components in an RNase-free tube. Mix the above mixture by quick vortex, then incubate 10 min at 25 °C. Incubate for 60 min at 47 °C. Stop the reaction by heating at 85 °C for 5 min. Chill on ice or at 40 °C. To perform PCR, add the finished RT reaction up to 1.5 of the final PCR volumes. The particular human specific primers for JAK-2, JAK-3, Stat-3 and GAPDH were used as internal control in this study. The primer sequence is exhibited in Table 3.

### 4.5. Quantitative Real-Time PCR

The experimental reaction was performed by adding the components in this order: sybergreen master mix 2× (12.5 μL Vol./reaction, 1×) with gene specific primers and cDNA (1 μL); total volume is 25 μL. The reaction microtubes were placed in the instrument, and the appropriate program according to the manufacturer’s instructions was run, (1 Cycles, 15 min Duration per cycle, 95 °C Temperature), and (40 Cycles, 30 s duration per cycle, 95 °C Temperature, and 30 s duration cycle at 61.5 °C Temperature). The analysis of fold change for JAK-2, JAK-3, and Stat-3 involved normalizing the data against reference genes that are consistently expressed. This normalized data was then compared to the untreated controls, known as the calibration sample, using the equation: 2−ΔΔCT, where ΔΔCT is calculated as (CT-target 2212 CT-reference)treated-sample—(CT-target—CT-reference)calibrator sample. The calibrator sample, which reflects the expression level (1×) of the target gene normalized to the reference gene, was selected from individuals with benign breast disease and assigned a relative expression value of 1 [23].

### 4.6. Protein Collection and Secondary Structure Prediction

Melittin, the 26-amino acid peptide found in *Apis mellifera* venom (GenBank: AFI40556.1), was selected for interaction with the human investigated proteins involved in breast cancer-related apoptosis. Additionally, we retrieved the sequences of the human proteins from the GenBank database, including JAK2 (GB: NP_001309125.1), JAK3 (GB: AAC50950.1), and STAT3 (GB: NP_644805.1). To predict the secondary structures of these proteins, we employed the SWISS-MODEL homology modeling server (https://swissmodel.expasy.org/, accessed on 2 February 2025) [24]. This approach utilizes comparative modeling techniques to generate secondary structure models based on evolutionary relationships between the target sequence and the template structures of known proteins. The models were further validated using Ramachandran plots and root-mean-square deviation (RMSD) calculations to ensure structural reliability. Furthermore, molecular docking studies were performed to analyze the binding affinities and interaction patterns between Melittin and the target proteins, providing insights into their potential roles in apoptosis regulation.

### 4.7. Protein Docking Prediction

Docking simulations between each protein of the *Homo sapiens* proteins and the *Apis mellifera* mature Melittin protein were performed using the LZerD web server (https://lzerd.kiharalab.org/, accessed on 2 February 2025) Venkatraman et al. 2009 [25]. LZerD employs an efficient and reliable algorithm for protein–protein docking, facilitating the exploration of potential binding modes and interactions between the proteins of interest. The docking algorithm was executed with default parameters, and a grid-based scoring function was employed to evaluate the binding affinity of different protein conformations. This enables the assessment of the structural compatibility and potential binding affinities between desired proteins using evaluation stats, including GOAP, DFIRE, and IT scores [26,27,28]. Following the generation of docked complex models, the top 50,000 models, ranked by LZerD shape score, were selected and subjected to clustering using a root-mean-square deviation (RMSD) threshold of 5 Å. Subsequently, cluster centers were scored using the ranksum procedure, as described in the server scorer section. Finally, the top 10 models were advanced to the refinement protocol. The top-ranking docking poses were selected for further analysis, considering both the energetic and geometric criteria. The root-mean-square deviation (RMSD) values were calculated using PyMol Ver 2.0. a widely used molecular visualization software [29]. PyMol offers robust tools for structural analysis and allows for the precise measurement of RMSD, providing insights into the degree of structural similarity between the docked complexes and their respective crystallographic or experimental structures. This analysis facilitates the evaluation of the accuracy and reliability of the predicted protein–protein interactions, aiding in the interpretation of docking results and the identification of potential binding modes with biological relevance.

### 4.8. Statistical Methods

All statistical analysis was performed with GraphPad Prism 4 software (Version 4.03, GraphPad Software, Inc., San Diego, CA, USA). Group differences in Figure 2, Figure 3, Figure 4 and Figure 5 were analyzed using one-way analysis of variance (ANOVA). Lower and upper bounds of the confidence intervals for individual experiments influence subgroup effects were evaluated. Data were analyzed using SPSS software (Version 21). We performed receiver–operator characteristic (ROC) and area under the curve (AUC) analyses for JAK2, JAK3 and STAT3 gene expression values. The data values were pooled between three individual experiments for wound healing assay and PCR experiments. Data are presented as mean ± SE. A value of *p* < 0.05 was considered statistically significant.

## 5. Conclusions

This study provides robust evidence of the synergistic anti-cancer effects of Melittin and Erlotinib in non-small cell lung cancer (NSCLC), highlighting their ability to significantly inhibit A549 cell proliferation and migration. The molecular docking analysis demonstrated that Melittin preferentially binds to key components of the JAK–STAT pathway, particularly JAK2 and JAK3, leading to the suppression of STAT3 activation and disruption of critical cancer-promoting pathways. These findings underscore the potential of Melittin as an effective adjuvant in cancer therapy, paving the way for future in vitro and in vivo studies to validate its therapeutic potential and explore its integration with existing treatments to enhance clinical outcomes.

## Figures and Tables

**Table 1:** Modeling the secondary structures of investigated proteins using SWISS-MODEL server.

Protein Name	Template	% Identity	Oligo-state	Folding	Coverage
Mel	P0DPR9.1.A	96.67%	Monomer	AlphaFold v2	100%
JAK2	O60674.1.A	100%	Monomer	AlphaFold v2	100%
JAK3	P52333.1.A	99.38%	Monomer	AlphaFold v2	100%
STAT3	P42227.1.A	99.87%	Monomer	AlphaFold v2	100%

**Table 2:** Predicted binding affinities between Melittin and JAK2, JAK3, and STAT3.

P_P Interaction	GOAP Score	GOAP Rank	DFIRE Score	DFIRE Rank	ITScore Score	ITScore Rank	Ranksum Score	RMSD
Mel-JAK3	−150,685	1	−107,543	9	−54,966	9	19	5.3
Mel-JAK2	−157,299	15	−110,823	24	−57,837	15	54	3.2
Mel-STAT3	−118,943	41	−72,978	88	−38,027	15	144	4.1

**Table 3:** Gene specific primers for mRNA expression studies.

S. No.	Primer Name	Forward Sequence	Reverse Sequence	PCR Products
1	Jak-2	AACGCTGAGGGGGATTATCT;	TGGTTGGGTGGATACCAGA	156
2	Jak-3	GGGAGATCCAGATCCTCAAAG;	GCAGATCTGTGAGGCGTAGAG	194
3	STAT-3	CTTTGAGACCGAGGTGTATCACC	GTCAGCATGTTGTACCACAGG	187
4	GAPDH	GTCTCCTCTGACTTCAACAGCG	ACCACCCTGTTGCTGTAGCCAA	166

## Data Availability

Available from the corresponding author upon request.

## Abbreviations

The following abbreviations are used in this manuscript:

SCLC	Non-Small Cell Lung Cancer
EGFR	Epidermal Growth Factor Receptor
µM	micromolar
JAK2	Janus kinase 2
JAK3	Janus kinase 3
